# The First Cry2Ac-Type Protein Toxic to *Helicoverpa armigera*: Cloning and Overexpression of *Cry2ac7* Gene from SBS-BT1 Strain of *Bacillus thuringiensis*

**DOI:** 10.3390/toxins9110358

**Published:** 2017-11-03

**Authors:** Faiza Saleem, Abdul Rauf Shakoori

**Affiliations:** 1 School of Biological Sciences, University of the Punjab, Quaid-i-Azam Campus, Lahore 54590, Pakistan; zoologist1pk@yahoo.com; 2 Department of Biotechnology, Lahore College for Women University, Lahore 54590, Pakistan

**Keywords:** *Bacillus thuringiensis* (*Bt*), cry2Ac7 gene, cloning, protein expression, bioassays, *Helicoverpa armigera*, *Musca domestica*

## Abstract

The Cry (crystal) proteins from *Bacillus thuringiensis* are known to have toxicity against a variety of insects and have been exploited to control insect pests through transgenic plants and biopesticides. *B. thuringiensis* SBS BT-1 carrying the *cry2* genes was isolated from soil samples in Pakistan. The 2-kb full length *cry2Ac* gene was cloned, sequenced, and submitted to the EMBL DNA database (Accession No. AM292031). For expression analysis, *Escherichia coli* DH5α was transformed with the fragment sub-cloned in pET22b expression vector using *Nde*I and *Hin*dIII restriction sites, and later confirmed by restriction endonuclease analysis. To assess the toxicity of Cry2Ac7 protein against lepidopteran and dipteran insects, BL21 (codon plus) strain of *E. coli* was further transformed with the recombinant plasmid. The 65-kDa protein was expressed in the form of inclusion bodies up to 180 OD units per liter of the medium. Inclusions were washed with a buffer containing 1.5% Triton-X 100 and >90% pure Cry2Ac7 was obtained. The inclusion bodies were dissolved in 50 mM K_2_CO_3_ (pH 11.5), dialyzed, and freeze-dried. This freeze-dried protein as well as inclusion bodies were used in bioassays against larvae of *Helicoverpa armigera* and *Musca domestica*. The freeze-dried protein was toxic to *H. armigera* larvae with an LC_50_ value of 131 ng/mL. However, Cry2Ac7 produced in *E. coli* did not show any mortality to *M. domestica* larvae. This is the first report of Cry2Ac protein toxic to *H. armigera*.

## 1. Introduction

*Bacillus thuringiensis* (*Bt*) is an aerobic, Gram-positive, endospore-forming soil bacterium. It produces parasporal protein toxins called insecticidal crystal proteins (Cry and Cyt proteins) or δ-endotoxins during the sporulation phase. These proteins are toxic to dipteran, lepidopteran, and coleopteran larvae [1]. They are also toxic to some hymenopteran, homopteran and mallophaga insects, as well as to many nematodes, flat worms, and Sarcomastigophora [2]. Despite the actual or presumed presence of various pathogenicity factors, *Bt* does not have a significant history of mammalian pathogenicity [3].

More than 250 crystal (Cry) proteins have been described [4,5]. The genes encoding Cry proteins are found (often clustered) on transmissible plasmids and flanking transposable elements, which explains their easy spread within the species [6,7,8]. Conjugation between different strains has been observed in soil environments as well as within the insects [9]. Individual Cry toxins have a defined spectrum of insecticidal activity, usually restricted to a few species within one particular order of insects. A few toxins have an activity spectrum that spans two or three insect orders—most notably Cry1Ba, which is active against larvae of moths, flies, and beetles [10], and Cry2Aa which is toxic to dipteran as well as lepidopteran insects [11]. 

*cry2A* gene encodes a protein of 65-kDa, which forms cuboidal crystals [11,12]. Three *cry2A* genes viz., *cry2Aa* [11,13], *cry2Ab* [11,14,15], and *cry2Ac* [15], have been reported. Cry2Aa is toxic to lepidopteran and dipteran larvae, while Cry2Ab and Cry2Ac are toxic only to lepidopteran species. *cry2Aa* and *cry2Ac* genes have a common characteristic in that these are placed at the third position in a three-gene operon. However, the crystallization of Cry2Aa protein requires the second gene, *orf2*, in the *cry2Aa* operon [16], while the two *orf*s upstream of *cry2Ac* gene do not have an apparent role in the formation of Cry2Ac inclusions [15]. On the other hand, *cry2Ab* gene is cryptic [11,14,17]. Dankocsik et al. [14] found Cry2Ab protein to be highly toxic to *Lymantria dispar*, *Heliothis virescens*, and *Trichoplusia ni*, but not toxic to *Aedes aegypti*. *cry2Ac* gene encodes approximately 65-kDa protein, which targets lepidopteran as well as dipteran insects. In the mature toxin, the N-terminal domain (residues 1–272) is a pore-forming seven-helical bundle. The second domain (residues 273–473) is a receptor binding β prism which is a three-fold symmetric arrangement of β sheets, each with a Greek key fold. The third domain (residues 474–623) is implicated in determining both larval receptor binding and pore function, and is a lectin-like C-terminal β sandwich. Cry2A binds to different receptors on BBMV, and thus is used for resistance management. Transgenic tobacco, rice, and cotton plants expressing *cry2A* genes have been generated for auto-protection against insects. *Bt* cotton that expresses two different *Bt* toxins (Cry1Ac + Cry2A) is being used to manage insect resistance [18]. Kota et al. [19] demonstrated that overexpression of the *Bt* Cry2Aa2 protein in chloroplasts confers resistance to plants against susceptible and *Bt*-resistant *Heliothis virescens*.

Besides their long-term use as a biological insecticide in the form of sprays of spore-crystal mixtures, individual Cry toxins have been expressed in transgenic plants to render crops resistant to insect pests. Since Cry1-type toxins have extensively been used in transgenic plants, there are reports that insects have developed resistance against some of these toxins. Akhurst et al. [20] reported the resistance of pests against *Bt*-cotton expressing Cry1Ac. In Pakistan, Maqbool et al*.* [21] generated transgenic rice *indica* expressing *cry2A* gene, which was reported to be effective against two major rice pests in the Indian subcontinent—the yellow stem borer and the rice leaf folder. Later, Zaidi [22] produced a transgenic tobacco plant, *Nicotiana tabacum* with *cry2A* to protect it against *Heliothis virescens*.

However, information about the distribution of *cry* genes is still limited and does not cover many distinct geographic areas. There is, therefore, a need to search for novel and more potent strains with new pathogenic spectra and wider host ranges, especially in parts of the world that have not been adequately sampled. Pakistan constitutes one such area which needs to be explored for crystalliferous strains with wider host ranges in this region. The present report characterizes *cry2A*-type gene(s) from these strains, and evaluates the toxicity of Cry2A proteins against the target insects, *Helicoverpa armigera* and *Musca domestica*.

## 2. Results

### 2.1. Genotyping for *cry2* Gene

All of the 50 local isolates were subjected to genotyping for *cry1*, *cry2*, and *cry4* genes using a universal set of primers (Supplementary Table S1). Only six isolates (SBS BT1-6, Supplementary Table S2) amplified 0.5 kb PCR product, which showed the presence of *cry2* gene in these isolates, while five of the locally isolated *Bt* isolates (CMBL BT1-5), obtained from Cell and Molecular Biology Lab (CMBL), University of the Punjab, Lahore, revealed the presence of *cry2* gene [23]. SBS BT1 was found negative for *cry1*, *cry1Ac*, and *cry4*, but positive for *cry2Ab* and *cry2Ac*.

### 2.2. Sequencing of the *cry2ac7* Gene

Full length *cry2Ac* gene, cloned from local isolate SBS-BT1, was sequenced from both strands and deposited to EMBL with accession number AM292031. It has now been named as Cry2Ac7 protein by *B. thuringiensis* Delta-Endotoxin Nomenclature Committee, becoming a new member of the *Bt* toxins (http://www.lifesci.susx.ac.uk/home/Neil_Crickmore/Bt/).

### 2.3. Hyperexpression of *cry2ac7* Gene in Escherichia coli (BL21 Codon Plus)

**Figure 1:**
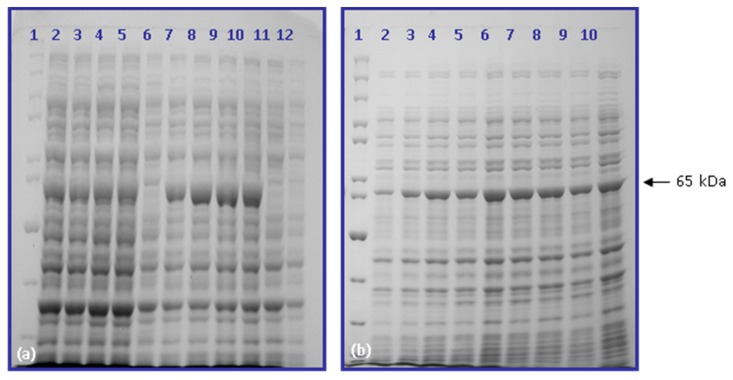
Optimization of Cry2Ac7 expression in BL21 (codon plus) (**a**) Molecular weight marker (lane 1), induction with 2.0 mM, 1.5 mM, 1.0 mM, 0.5 mM IPTG at 37 °C (lanes 2–5), uninduced sample (lane 6), induction with 2.0 mM, 1.5 mM, 1.0 mM, 0.5 mM at 25 °C (lanes 7–10), induced and uninduced pET22b (lanes 11 and 12). (**b**) Molecular weight marker (lane 1), induction with 0.5 mM IPTG at 16 °C for 2, 4, 6, and 8 h (lanes 3, 5, 7, and 11), induction with 0.5 mM IPTG at 25 °C for 0, 2, 4, 6, and 8 h (lanes 2, 4, 6, 8, and 10).

For overexpression, BL21 codon plus strain of *E. coli* was transformed with the recombinant *SBS-BT1/cry2Ac7*. Ten colonies were picked up from the plate, inoculated in LB broth and induced with IPTG (1 mM) at 37 °C for 8 and 20 h. Protein profiles were analyzed with SDS-PAGE. All colonies expressed about 65 kDa Cry2Ac7 protein equally after 8 h. Furthermore, the IPTG concentration was optimized and no visual difference was noted in the expression of Cry2Ac7 when induced with 0.5, 1.0, 1.5, and 2.0 mM IPTG concentration (Figure 1a). Also, the incubation temperature of 25 °C was found to be optimum for the expression with varying concentrations of IPTG (Figure 1b), though the expression of non-target proteins was also found low when induced at 16 °C. 

### 2.4. Partial Purification of Cry2Ac7 Inclusion Bodies

**Figure 2:**
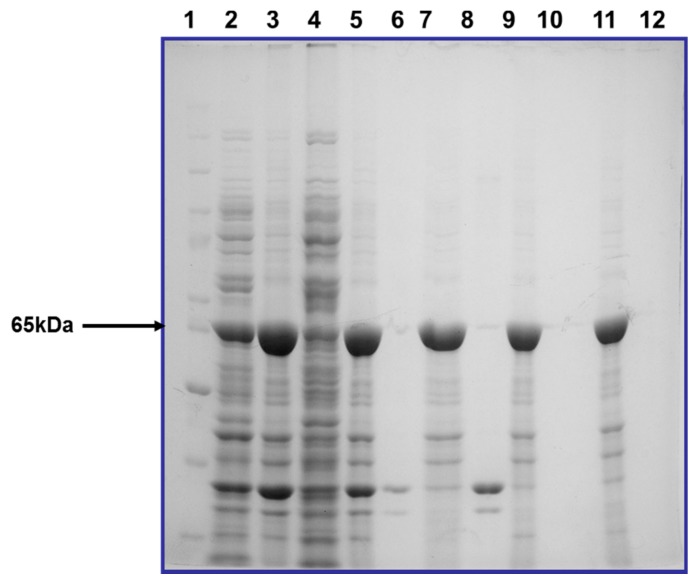
Purification of inclusion bodies of Cry2Ac7 expressed in BL21 (codon plus) induced with 0.5 mM IPTG at 25 °C for 4 h: Molecular weight marker (lane 1), total cell protein (lane 2), pellet 1 (lane 3), supernatant 1 (lane 4), pellet 1 (lane 5), supernatant 2 (lane 6), pellet 3 (lane 7), supernatant 3 (lane 8), pellet 4 (lane 9), supernatant 4 (lane 10), pellet 5 (lane 11), supernatant 5 (lane 12).

The Cry2Ac7 inclusion bodies were collected from disrupted cells after centrifugation. They were partially purified by washing with different buffers as mentioned in the methodology. The SDS-PAGE analysis after each step revealed the removal of unwanted proteins from the inclusions (Figure 2).

### 2.5. Toxicity Assay of Cry2Ac7 Toxin against Helicoverpa armigera

**Figure 3:**
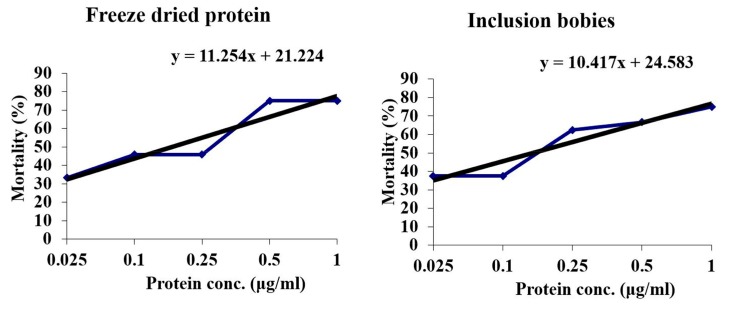
Bioassays of *Helicoverpa armigera* with Cry2Ac7 protein, in the form of freeze-dried powder and inclusion bodies. Mortality was determined using Probit analysis [24].

**Figure 4:**
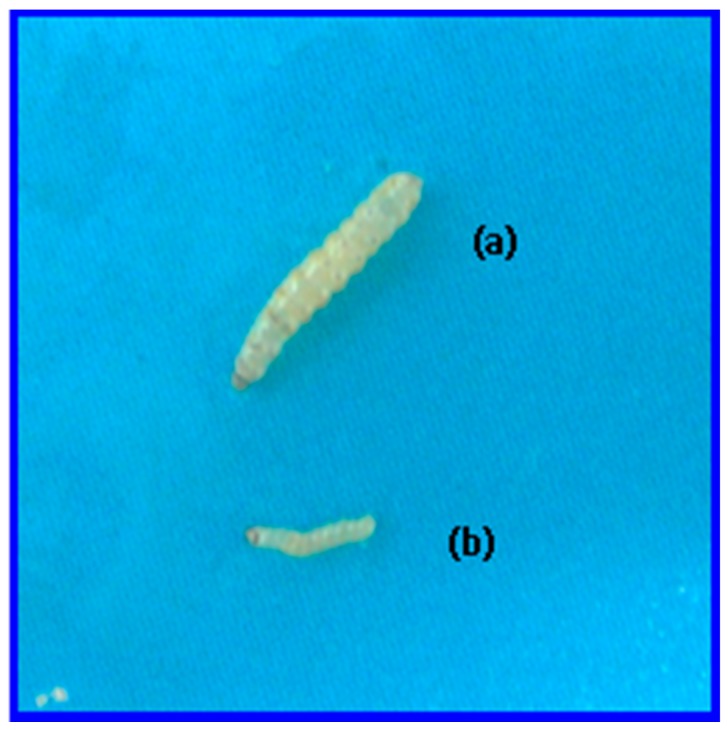
Growth difference in first instar larvae of *H. armigera* after feeding for four days on normal synthetic diet (**a**) and on diet with Cry2Ac7 protein (**b**).

Cry2Ac7 protein, in the form of partially purified freeze-dried powder as well as crude inclusion bodies, was used in toxicity assays against *H. armigera*. Both forms of the proteins were almost equally toxic to the insects with an LC_50_ value of 131 ng/mL (0.131 µg/mL) for freeze-dried protein and 118 ng/mL (0.118 µg/mL) for inclusion bodies, as shown in Figure 3. The insects stopped feeding within 24 h. Mortality started after 72 h. Though a few insects resisted even high protein dose, their sizes were considerably smaller than the control insects (Figure 4).

In *M. domestica*, the protein had no toxic effect on the growth and vigor of the emerging flies (data not included).

It may, however, be kept in mind that the inclusions produced in *E. coli* are not reflective of the true nature of the crystals, and the bioassay data reported here for *H. armigera* and *M. domestica* may not be interpretable and comparable with other Cry2A-based assays in which native Cry2A crystal/spore mixtures have been used.

## 3. Discussion

### 3.1. Profile of Cry2A Subtypes and Toxicity

SBS-BT1 indeed showed the toxicity against lepidopteran (*H. armigera*) and dipteran (*M. domestica*) insects. PCR amplification, a sensitive method which can rapidly detect and identify target DNA sequences, had been employed by researchers to identify *Bt* genes [4,6]. In this study, the technique was used to investigate SBS-BT1 for the presence of the *cry1, cry1Ac, cry2,* and *cry4* genes. The results indicated the presence of *cry2* gene only, whereas *cry1* and *cry4* were not detected. The toxicity of the strain in the bioassays was therefore possibly because of *cry2* gene(s). There are however, a number of other factors apart from Cry toxins in *Bt* which show insecticidal activity found within the crystals or act as enhancers for Cry or cytolysins (Cyt) toxins. *Bt* produces various virulence factors other than the crystal proteins, including secreted insecticidal protein toxins, α-exotoxins, β-exotoxins, hemolysins, enterotoxins, chitinases, and phospholipases [25]. The spore itself contributes to pathogenicity, often synergizing the activity of the crystal proteins. In the presence of so many factors contributing to pathogenicity, the exact contribution of each factor is often unknown [6]. 

Genotyping with specific primers of *cry2* subfamilies indicated the presence of *cry2Ab* and *cry2Ac* genes. These *cry2* gene(s) might be the target gene-encoding protein toxic to the tested insects. To the best of our knowledge, this is the first report of Cry2Ac-type protein toxic to American bollworm, *H. armigera*. Wu et al. [15] reported the low toxicity of Cry2Ac1 toxin (accession #CAA40536) having an LC_50_ value of 4000 ng/mL, while Cry2Ac7 is 30.5-fold more toxic with an LC_50_ value of 131 ng/mL. 

### 3.2. Sequence Homology of Cry2Ac7 Protein with Other Related Proteins

Cry2Ac7 has 97.43% amino acid homology with Cry2Ac1. There are 16 amino acid differences between the two proteins (Table 1 and Table 2). Most of these differences lie in Domain I, which is involved in membrane insertion and pore formation, and Domain II, which is involved in receptor recognition and binding. Therefore, the increased toxicity of Cry2Ac7 may be due to all or some of these altered amino acids. None of the rest of Cry2Ac-type protein has been tested against this particular insect.

Interestingly, the difference of a few amino acids can alter the toxicity spectrum of Cry proteins. Widner and Whiteley [26] studied the host range specificity of two highly related crystal protein genes from *Bacillus thuringiensis* subsp. *kurstaki* HD-1, designated *cry2Aa* and *cry2Ab* (previously named *cryIIA* and *cryIIB*, respectively). Their respective gene products are 87% identical but exhibit different toxicity spectra; Cry2Aa is toxic to both mosquito and tobacco hornworm larvae, whereas Cry2Ab is toxic only to the latter. They generated hybrids of the *cry2Aa* and *cry2Ab* genes as well as their resultant gene products were assayed for toxicity. A short segment of Cry2Aa corresponding to residues 307 through 382 was shown to be sufficient for altering host range specificity i.e., when this region replaced the corresponding segment of Cry2Ab, the resulting hybrid protein acquired toxicity against mosquitoes. The Cry2Aa and Cry2Ab polypeptides differ by only 18 amino acids in this region, indicating that very few amino acid changes can have a substantial effect on the toxicity spectra of these proteins.

On the other hand, the toxicity of Cry2Ac5 (Accession # DQ341379) was reported to the third instar larvae of mosquito, *A. albopictus* [27]. Cry2Ac7 and Cry2Ac5 are 99.2% identical and differ only in seven amino acids (Table 1 and Table 2). Five of these differences lie in Domain I, which is involved in membrane insertion and pore formation, whereas two amino acid differences lie in Domain III. Domains II and III are both involved in receptor recognition and binding. Additionally, a role for domain III in pore function has been found [3]. The *cry2Ac5* encoded a toxic protein against *A. albopictus*, which was not in agreement with the previous reports that Cry2Ab, Cry2Ac, and Cry2Ad were only toxic to lepidopteran insects [15,28]. It might also be possible that the interesting activity was due to the fact that the other Cry2Ac toxins were not tested against this particular species of mosquito. Further analysis of the differences in the amino acid sequence and the level and host range of toxicity is in progress, and would be the subject of further and potentially more extensive study.

**Figure 5:**
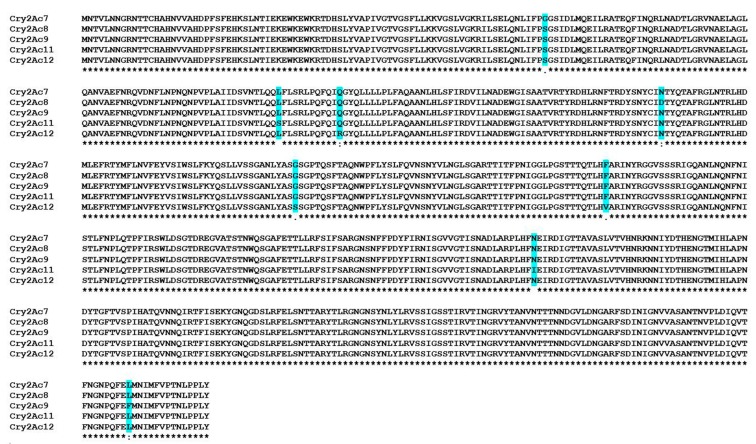
Multiple sequence alignment of Cry2Ac7 protein with Cry2Ac8, Cry2Ac9, and Cry2Ac12 reported from Pakistani isolates. Cry2Ac11 (HD29) is the positive control.

Figure 5 shows the multiple sequence alignment of Cry2Ac7 protein with Cry2Ac8, Cry2Ac9, and Cry2Ac12 proteins reported from Pakistani isolates. Although the samples were collected from different parts of Pakistan over a period of six years, 99.5% amino acid homology was found amongst these proteins. There was lesser homology with rest of the Cry2Ac-type toxins from other geographical regions of the world (homologies not shown here). This high degree of similarity among the toxins from one geographical region suggests some geographical basis for the origin and evolution of these toxins. These proteins contain at least one unique variation in the amino acid sequence. Almond and Dean [29] showed that even a single amino acid change can dramatically reduce the stability of Cry proteins and hence affect the toxicity of toxins. Further studies are needed to analyze amino acid variations which are crucial in assigning toxicity spectra to Cry2Ac-type toxins. 

## 4. Materials and Methods

### 4.1. Sampling and Isolation of *Bacillus thuringiensis* Strains

Soil samples were collected from different areas of Lahore, Pakistan. The samples were processed by the acetate selective method [30]. One gram of each sample was incubated for 4 h at 30 °C and 150 rpm in a conical flask containing 20 mL of 0.30 M sodium acetate (pH 6.8). This selectively suppressed *Bt* spores that germinated when plated on a rich agar medium after incubating 2 mL of the sample at 80 °C for 10 min. Three hundred microliters of each heat-treated sample was then spread-plated and incubated at 30 °C for 24 h on plates of a medium containing (per liter) 3 g tryptone, 2 g tryptose, 1.5 g yeast extract, 0.05 M sodium phosphate (pH 6.8), 0.005 g MnCl, and 15 g agar. Developing colonies were grown at 30 °C for 24 h on LB agar plates (1 g tryptone, 0.5 g yeast extract, 0.5 g NaCl. and 1.5 g bacteriological agar per 100 mL of water). Smears of these were stained with Gram’s staining. Diagnostic schemes given in Bergey’s Manual of Determinative Bacteriology [31] were followed for the identification of different *Bt* subspecies.

### 4.2. Ribotyping

Full length 16S rDNA (1692 bp) was amplified through PCR [32] and cloned in pTZ57R/T vector. These were further proceeded for sequencing. Then, 1.6 kb fragment was sequenced from both strands with internal primers (Supplementary Table S3) and submitted to the DNA database (EMBL AM 778998).

### 4.3. DNA Isolation and Genotyping

Total cell DNA of *Bt* was prepared according to Kronstad et al. [33]. Cells grown in 500 mL of Spizizen medium ((NH_4_)_2_SO_4_ 2 g, KH_2_PO_4_ 6 g, sodium citrate.2H_2_O 1 g, MgSO_4_·7H_2_O 0.2 g, glucose 0.5%, K_2_HPO_4_·3H_2_O 18.3 g, tryptone 20 g, yeast extract 5 g, dissolved per liter of water) in a 2.8-L flask with shaking at 37 °C. Cells were harvested at an optical density of 0.7 at 600 nm. The cultures were harvested by centrifugation at 6000 rpm (4355 rcf) for 10 min at 4 °C in a Beckman centrifuge, and finally washed with 100 mL of a solution containing 100 mM NaCl, 10 mM Tris (pH 7.9), and 10 mM EDTA. The bacterial pellet was resuspended in 5 mL of a solution containing 150 mM NaCl and 100 mM EDTA at pH 7.9. Lysozyme was added to give a final concentration of 0.25 mg/mL, and the preparation was incubated at 37 °C for 20 min. To lyse the cells, 6.25 mL of a third solution (100 mM Tris (pH 7.9), 100 mM NaCl, 2% SDS) was added. The preparation was mixed gently by inverting the tube four or five times and incubated at 60 °C until clear (usually 30–45 min). The lysate was then extracted four times with phenol-chloroform (1:1), which had been equilibrated with the above Tris-NaCl-SDS solution. The aqueous phase was extracted each time with a wide-bore pipette. After the final extraction, cold ethanol was added and the DNA was spooled out with a glass rod. The DNA was then rinsed with 70% ethanol, dissolved in TE buffer (10 mM Tris (pH 7.9), 1 mM EDTA) and stored in aliquots at −20 °C. Universal set of primers [4,34] was used to detect *cry1*, *cry2*, *cry4*, *cry1Ac*, *cry2Aa*, *cry2Ab*, and *cry2Ac* genes in these isolates (Supplementary Table S2). 

### 4.4. Cloning and Sequencing of the *cry2ac7* Gene

To amplify full length gene from the local *Bt* isolates, a primer pair was designed manually from the pre-existing sequences of *cry2Ac* gene from DNA databases. PCR reaction mixture (50 µL) containing *Taq* buffer 1x, MgCl_2_ 1.5 mM, dNTPs 200 µM, each of the primers 50 pmol, DNA 0.2–0.8 µg, and *Taq* DNA polymerase 2.5–5.0 units, was run on an Applied BioSystems 2720 thermal cycler. The thermal cycle of the reaction consisted of pre-PCR heating at 94 °C for 5 min, a final extension at 72 °C for 10 min, and 30 cycles each of denaturation at 94 °C for 1 min, annealing at 50 °C for 0:45 min, and an extension at 72 °C for 2 min. For sub-cloning in expression vector pET22b, *cry2Ac* gene was amplified with the following primer pair using T/A cloned gene as a template:

pET21a-N   5′  GCTCGGTACCTCGCTGAATGCATCTAGCATATGAATAC 3′
M13 R           5′-d(GAGCGGATAACAATTTCACACAGG)-3′)

*Nde*I-*Hin*dIII digested vector and PCR fragment were used in 1:3 ratio in a 50-µL reaction containing 5 µL of 10x ligation buffer and 5 units of T4 DNA ligase. The reaction mixture was incubated at 22 °C overnight. BL21 (codon plus) strain of *E. coli* was transformed with the recombinant vector for expression analysis.

### 4.5. Sub Cloning in Expression Vector pET22b

*cry2ac7* gene was amplified with N-terminal primer containing *Nde*I restriction site and ligated in expression vector pET22b to form a recombinant p*SBS-BT1/cry2Ac7*. DH5α was transformed with this recombinant DNA. Six colonies were picked up from the plate. All of them had recombinant plasmids, which was confirmed by plasmid miniprep and restriction analysis of the plasmid. 

### 4.6. Expression of *cry2ac7* Gene in Escherichia coli (BL21C^+^)

BL21 codon plus (BL21C^+^) was transformed with the recombinant plasmid p*SBS-BT1/cry2Ac7*. A single colony was inoculated in LB broth containing 100 μg/mL ampicillin and grown at 37 °C overnight with continuous shaking. The next day, it was seed inoculated (1%) in LB broth (ampicillin 100 μg/mL), and grown at 37 °C for about 2 h (until OD_550_ was 0.6–0.8) with continuous shaking. It was induced with various concentrations of IPTG (0.5, 1.0, 1.5, and 2.0 mM) at varying temperatures (16 °C, 25 °C, and 37 °C) for different time periods (2, 4, 6, 8, and 18 h). Expression levels of the protein were analyzed on 12% SDS-PAGE. In all experiments, BL21C^+^ transformed with non-recombinant pET22b plasmid was proceeded in parallel as a negative control. 

### 4.7. Purification of Cry2Ac7

The *E. coli* cells, expressing the recombinant protein in the form of inclusion bodies, were harvested and resuspended in wash buffer (Tris-Cl (pH 8.0) 50 mM, Triton X-100 1.5%, NaCl 200 mM, EDTA (pH 8.0) 10 mM, DTT 2 mM). The cells were lysed by passing through a French press under 2500 psi internal pressure and then centrifuged at 9500 rpm (15,971 rcf) for 15 min at 4 °C to harvest inclusion bodies. The pellet was washed once again with wash buffer, twice with distilled water, once with 0.5 M NaCl, and twice with water. The pellet was resuspended in 50 mM K_2_CO_3_ (pH 11.5) and incubated at 37 °C for 1.5–2 h. It was then centrifuged at 9500 rpm (15,971 rcf) for 25 min at 4 °C to harvest the insoluble inclusions. The solubilized part was dialyzed against different decreasing concentrations of K_2_CO_3_ (25 mM, 10 mM, 5 mM, 2.5 mM, 1 mM) and finally against distilled water overnight at 4 °C. The protein was then freeze-dried.

The freeze-dried protein was solubilized in 20 mM Tris (pH 10.6) and dialyzed against the same buffer overnight at 4 °C, centrifuged at 6000 rpm (4468 rcf) to remove any particulate matter, then filtered through 0.4-µm Millipore filter and injected in the Amersham Bioscience FPLC machine. Fractions were analyzed on SDS-PAGE and protein was quantified by taking OD_280_ (1 OD unit = 1 mg) as well as by a modified Lowry method [35].

### 4.8. Toxicity Assay of Cloned Toxin against *H. armigera* and *M. domestica*

Various concentrations of Cry2Ac7 protein (0.025 to 1.00 µg/g for *H. armigera* and 0.217 to 8.696 µg/g for *M. domestica)*, in two separate experiments, were thoroughly mixed in the artificial diet of *H. armigera* [36] and *M. domestica* [37]. In a negative control, the recombinant protein was not added. 

Eight to 10 neonate larvae of *H. armigera* were placed over their diet surface to feed ad libitum, each in a separate vial. The vials were covered with aluminum foil and kept at 24 ± 2 °C. Mortality was recorded after 72 h. Likewise, 50 eggs of *M. domestica* were placed on the diet surface for the eggs to hatch within 8–16 h and the larvae were allowed to feed on the diet ad libitum at 26 ± 2 °C. The mortality was recorded after 10–13 days. All experiments were conducted in three replicas. 

### 4.9. Statistical Analysis

The mortality was determined using Probit analysis [37].

## Figures and Tables

**Table 1:** CLUSTALW (2.1) multiple sequence alignment of Cry2Ac1, Cry2Ac7, and Cry2Ac5 proteins.

y2Ac5	MNNVLNSGRNTTCHAHNVVAHDPFSFEHKSLNTIEKEWKEWRRTDHSLYVAPIVGTVGSF
Cry2Ac7	MNTVLNNGRNTTCHAHNVVAHDPFSFEHKSLNTIEKEWKEWKRTDHSLYVAPIVGTVGSF
Cry2Ac1	MNTVLNNGRNTTCHAHNVVAHDPFSFEHKSLNTIEKEWKEWKRTDHSLYVAPIVGTVGSF
	**.***.**********************************:******************
Cry2Ac5	LLKKVGSLVGKRILSELQNLIFPSGSIDLMQEILRATEQFINQRLNADTLGRVNAELAGL
Cry2Ac7	LLKKVGSLVGKRILSELQNLIFPGGSIDLMQEILRATEQFINQRLNADTLGRVNAELAGL
Cry2Ac1	LLKKVGSLVGKRILSELQNLIFPSGSIDLMQEILRATEQFINQRLNADTLGRVNAELAGL
	***********************.************************************
Cry2Ac5	QANVAEFNRQVDNFLNLNQNPVPLAIIDSVNTLQQLFLSRLPQFQIQGYQLLLLPLFAQA
Cry2Ac7	QANVAEFNRQVDNFLNPNQNPVPLAIIDSVNTLQQLFLSRLPQFQIQGYQLLLLPLFAQA
Cry2Ac1	QANVAEFNRQVDNFLNPNQNPVPLAIIDSVNTLQQLFLSRLPQFQIQGYQLLLLPLFAQA
	**************** *******************************************
Cry2Ac5	ANLHLSFIRDVILNADEWGISAATVRTYRDHLRNFTRDYSNYCINTYQTAFRGLNTRLHD
Cry2Ac7	ANLHLSFIRDVILNADEWGISAATVRTYRDHLRNFTRDYSNYCINTYQTAFRGLNTRLHD
Cry2Ac1	ANFNLSFIRGVILNADEWGISAATVRTYRDHLRKFHRDYSNYCINPYQTAFRGLNHRLPD
	**::*****.***********************:* *********.********* ** *
Cry2Ac5	MLEFRTYMFLNVFEYVSIWSLFKYQSLLVSSGANLYASGSGPTQSFTAQNWPFLYSLFQV
Cry2Ac7	MLEFRTYMFLNVFEYVSIWSLFKYQSLLVSSGANLYASGSGPTQSFTAQNWPFLYSLFQV
Cry2Ac1	MLEFRTYMFLNVFEYVSIWSLFKYQSLLVSSGANLYASGSGPTQSFTAQNWPFLYSLFQV
	************************************************************
Cry2Ac5	NSNYVLNGLSGARTTITFPNIGGLPGSTTTQTLHFARINYRGGVSSSRIGQANLNQNFNI
Cry2Ac7	NSNYVLNGLSGARTTITFPNIGGLPGSTTTQTLHFARINYRGGVSSSRIGQANLNQNFNI
Cry2Ac1	NSNYVLNGLSGARTTITFPNIGGLP-VYHNSTLHFARINYRGGVSSSRIGQANLNQNFNI
	************************* ..*****************************
Cry2Ac5	STLFNPLQTPFIRSWLDSGTDREGVATSTNWQSGAFETTLLRFSIFSARGNSNFFPDYFI
Cry2Ac7	STLFNPLQTPFIRSWLDSGTDREGVATSTNWQSGAFETTLLRFSIFSARGNSNFFPDYFI
Cry2Ac1	STLFNPLQTPFIRSWLDSGTDREGVATSTNWQSGAFETTLLRFSIFSARGNSNFFPDYFI
	************************************************************
Cry2Ac5	RNISGVVGTISNADLARPLHFNEIRDIGTTAVASLVTVHNRKNNIYDTHENGTMIHLAPN
Cry2Ac7	RNISGVVGTISNADLARPLHFNEIRDIGTTAVASLVTVHNRKNNIYDTHENGTMIHLAPN
Cry2Ac1	RNISGVVGTISNADLARPLHFNEIRDIGTTAVASLVTVHNRKNNIYDTHENGTMIHLAPN
	************************************************************
Cry2Ac5	DYTGFTVSPIHATQVNNQIRTFISEKYGNQGDSLRFELSNTTARHTLRGNGNSYNLYLRV
Cry2Ac7	DYTGFTVSPIHATQVNNQIRTFISEKYGNQGDSLRFELSNTTARYTLRGNGNSYNLYLRV
Cry2Ac1	DYTGFTVSPIHATQVNNQIRTFISEKYGNQGDSLRFELSNPTARYTLRGNGNSYNLYLRV
	****************************************.***:***************
Cry2Ac5	SSIGSSTIRVTINGRVYTANVNTTTNNDGVLDNGARFSDINIGNVVASANTNVPLDIQVT
Cry2Ac7	SSIGSSTIRVTINGRVYTANVNTTTNNDGVLDNGARFSDINIGNVVASANTNVPLDIQVT
Cry2Ac1	SSIGSSTIRVTINGRVYTANVNTTTNNDGVLDNGARFSDINIGNVVASANTNVPLDIQVT
	************************************************************
Cry2Ac5	FNGNPQFELMNIMFVPTNIPPLY
Cry2Ac7	FNGNPQFELMNIMFVPTNLPPLY
Cry2Ac1	FNGNPQFELMNIMFVPTNLPPLY
	******************:****

**Table 2:** Variation in the amino acid sequences of Cry2Ac1, Cry2Ac5, and Cry2Ac7 proteins.

Amino Acid	Cry2Ac1	Cry2Ac5	Cry2Ac7
3		Asn	Thr
7		Ser	Asn
42		Arg	Lys
84	Ser	Ser	Gly
137		Leu	Pro
183	Phe		Leu
184	Asn		His
190	Gly		Asp
214	Lys		Asn
216	His		Thr
226	Pro		Thr
236	His		Thr
239	Pro		His
326	Insertion		Gly
327	Val		Ser
328	Tyr		Thr
329	His		Thr
330	Asn		Thr
331	Ser		Gln
521	Pro		Thr
525		His	Tyr
619		Ile	Leu

## Supplementary Materials

The following are available online at www.mdpi.com/2072-6651/9/11/358/s1, Table S1: Universal set of primers used for genotyping of *Bt* isolates for *cry 1*, *cry2* and *cry 4* genes, Table S2: Varies local isolates of *B. thuringiensis* harboring *cry2* gene, Table S3: Internal primers used for sequencing full length genes.
