# Supplementary material for: The First Cry2Ac-Type Protein Toxic to Helicoverpa armigera: Cloning and Overexpression of Cry2ac7 Gene from SBS-BT1 Strain of Bacillus thuringiensis
Source: Toxins (Basel). 2017 Nov 3;9(11):358. doi: 10.3390/toxins9110358 (PMC5705973; doi:10.3390/toxins9110358)
Supplement: Supplementary file 1 [file toxins-09-00358-s001.pdf]

Supplementary Materials: The First Cry2Ac-Type Protein Toxic to *Helicoverpa armigera*: Cloning and Overexpression of *Cry2ac7* Gene from SBS-BT1 Strain of *Bacillus thuringiensis*

Faiza Saleem and Abdul Rauf Shakoori

Table S1. Universal set of primers used for genotyping of *Bt* isolates for *cry 1, cry2* and *cry 4* genes.

Sr. no.	Primer Pairs	Primer Sequence (5′→3′)	Gene Amplified	Reference
1	Un1(d)<br>Un1(r)	5′-CATGATTCATGCGGCAGATAAAC-3′<br>5′-TTGTGACACTTCTGCTTCCCATT-3′	*cry1* (Partial)	Ben-Dov et al. (1997)
2	Un2 (d)<br>Un2 (r)	GTTATTCTTAATGCAGATGAATGGG<br>CGGATAAAATAATCTGGGAAATAGT	*cry2* (Partial)	Ben-Dov et al. (1997)
3	Un4(d)<br>Un4(r)	5′-GTTATTCTTAATGCAGATGAATGGG-3′<br>5′-GCGTGACATACCCATTTCCAGGTCC-3′	*cry4* (Partial)	Ben-Dov et al. (1997)
4	1Ac F<br>1Ac R	5′ GTATGCTTCTGTAACCCCGATTCACCTC 3′<br>5′ CCTGCAGTCCCACTAAAATTTCTAACACCTACTA 3	*Cry1Ac* (Partial)	Alberola et al. (1999)
5	2Aa F<br>2Aa R	5′ GGATATTGAGTGAATTATGGGGGATA 3′<br>5′ CCGCTATAATTAACCCTGGCACTATTCAATGA 3	*Cry2Aa* (Partial)	Alberola et al. (1999)
6	2Ab F<br>2Ab R	5′ CACAGCAGACCCAATCATTTACTTCACAAGA 3′<br>5′ CTGTAAAAGCACCACTCCTTAACCCTAAA 3′	*Cry2Ab* (Partial)	Alberola et al. (1999)
7	2Ac F<br>2Ac R	GGAGTGTCATCTAGCCGCATAGGTCAAG<br>ACCATAATATTCATAAGCTCAAATTGTGGATTG	*cry2Ac* (Partial)	Alberola et al. (1999)
8	2AcT F<br>2AcT R	ATGAATACTGTATTGAATAACGGAAG<br>CCTTAATAAAGTGGTGGAAGATTAG	*cry2Ac* (Complete)	This study
9	67F<br>1671R	TGAAAACTGAACGAAACAAAC<br>CTCTCAAAACTGAACAAAACGAAA	16S rDNA (Complete)	Sacchi et al. (2002)

Table S2. Various local isolates of B. *thuringiensis* harboring *cry2* gene.

Sr. No.	Catalogue No.	PCR Based Detection			
		cry1	*cry1Ac*	*cry2*	*cry4*
1	HD29	+	-	Ab, Ac, Ad	-
2	CMBL-BT1	+	-	Aa, Ab, Ac	+
3	CMBL-BT2	+	-	Ab, Ac, Ad	+
4	CMBL-BT3	+	-	Ab, Ac	+
5	CMBL-BT4	+	-	Ac	+
6	CMBL-BT5	+	-	Ab, Ac, Ad	+
7	SBS-BT1	-	-	Ab, Ac	-
8	SBS-BT2	-	-	Aa	+
9	SBS-BT3	-	-	+	-
10	SBS-BT4	-	-	+	+
11	SBS-BT5	-	-	+	+
12	SBS-BT6	-	-	Ab, Ac	+

Table S3. Internal primers used for sequencing full length genes.

Serial No.	Internal Primers	Primer Sequence (5′→3′)	Gene Amplified	Reference
1	2Ac 1	GCACAGGCAGCCAATTTAC	All for sequencing *cry2Ac* (Partial)	This study
2	2Ac 2	GAGGTGGAGTGTCATCTAGC		This study
3	2Ac 3	GAGTGGCATGTATTGGAGATACGG		This study
4	2Ac 4	GTAAATTGGCTGCCTGTGC		This study
5	2Ac 5	GCTAGATGACACTCCACCTC		This study
6	2Ac 6	CCGTATCTCCAATACATGCCACTC		This study
7	2Ad 1	CCCTAACCGAAACGCTGTTCC	All for sequencing *cry2Ad* (Partial)	This study
8	2Ad 2	GGAGGAATTACATCTGGTAG		This study
9	2Ad 3	CCGGCGCTAAATGAATC		This study
10	2Ad 4	GGAACAGCGTTTCGGTTAGGG		This study
11	2Ad 5	CTACCAGATGTAATTCCTCT		This study
12	2Ad 6	GATTCATTTAGCGCCGG		This study
13	16S 1	GAAAGTCTGACGGAGCAACGC	All for sequencing *16S rDNA* (Partial)	This study
14	16S 2	GGTCTTGACATCCTCTGAC		This study
15	16S 3	GATCAAGGGTTGCGCTCGTTGC		This study
16	16S 4	GCGTTGCTCCGTCAGACTTTC		This study
17	16S 5	GTCAGAGGATGTCAAGACC		This study
18	16S 6	GCAACGAGCGCAACCCTTGATC		This study
